# Possible infectious causes of spontaneous splenic rupture: a case report

**DOI:** 10.1186/1752-1947-8-396

**Published:** 2014-11-30

**Authors:** Grace Y Lam, Adrienne K Chan, Jeff E Powis

**Affiliations:** 21 Department of Medicine, University of Toronto, Toronto, Canada; 22 Department of Infectious Diseases, Toronto East General Hospital, 825 Coxwell Ave, Toronto, ON M4C 3E7 Canada; 23 Dignitas International, Zomba, Malawi

**Keywords:** Atraumatic splenic rupture, *Bartonella henselae*, Epstein–Barr virus, Infectious diseases

## Abstract

**Introduction:**

Spontaneous atraumatic splenic rupture is a rare but dramatic occurrence that is most commonly attributed to infection or neoplasia. Deciphering the etiology can be challenging with many cases remaining unclear despite full investigation.

**Case presentation:**

We report the case of a previously healthy and immunocompetent 52-year-old Caucasian woman with a remote history of clinically diagnosed infectious mononucleosis who experienced sudden atraumatic splenic rupture after an untreated stray cat bite.

**Conclusions:**

The differential diagnosis for atraumatic splenic rupture, specifically its infectious causes, is reviewed. Key clinical and laboratory findings that differentiate *Bartonella henselae* infection and Epstein–Barr virus reinfection are reviewed.

## Introduction

Splenic rupture typically presents nonspecifically with left upper quadrant abdominal tenderness with or without distention, syncope, and a rapid drop in blood pressure. If severe, shock and alterations in level of consciousness may also be observed. Its diagnosis is most often established with ultrasonographic or computed tomography (CT) abdominal imaging. However, determining the etiology is often more difficult. The causes of splenic rupture can be generally divided into two categories – traumatic or atraumatic – where trauma explains the majority of cases. The diagnosis of atraumatic splenic rupture (ASR) can be made with the Orloff and Peskin criteria, which states that ASR can be diagnosed when the following four criteria are met: 1) thorough history reveals no antecedent trauma; 2) no evidence of disease in organs other than the spleen that can cause rupture; 3) no perisplenic adhesions or scarring consistent with trauma or past rupture; and 4) normal spleen on gross and histological examination [1].

The causes of ASR are varied and can be classified into seven main categories: neoplastic, infectious, hematological, inflammatory, iatrogenic, primary splenic causes or idiopathic (see Table 1) [2]. Proportionally, neoplasia and infection account for more than half of the cases [3].

**Table 1:** **Causes of non-traumatic spontaneous splenic rupture**

Neoplastic	Infectious	Hematologic	Inflammatory	Iatrogenic	Primary splenic disorder
- Leukemia	Viral	- Hemophilia	- Acute or chronic	- Heparin/ Warfarin	- Splenic cyst
- Lymphoma	- Epstein–Barr virus	- Factor XIII deficiency	Pancreatitis	- Granulocyte-colony stimulating factor	- Splenic angiomatosis
- Polycythemia vera	- Cytomegalovirus	- Protein S deficiency	- Primary amyloidosis	- Thrombolytic therapy	- Splenic peliosis
- Multiple myeloma	- Human immunodeficiency virus	- Idiopathic thrombocytopenic purpura	- Lupus erythematosus	- Dialysis	- Splenic infarctions or venous thrombosis
- Myelodysplastic disorders	- Hepatitis A/B/C	- Hemolytic anemia	- Rheumatoid arthritis	- Lithotripsy	- Portal hypertension
	- Rubella		- Polyarteritis nodosa		- Congenital malposition (i.e. short splenic pedicle)
	- Varicella				- Splenic malignancy
	Bacterial				
	- Legionellosis				
	- Bartonellosis				
	- Infective endocarditis (Staphylococcus, Streptococcus, Clostridium, Actinomycosis, Pseudomonas among the top causes)				
	Other				
	- Malaria				
	- Syphilis				
	- Toxoplasma				

Regardless of etiology, the immediate management of ASR can be varied, depending on the degree of splenic injury [4]. If the degree of splenic injury is mild, then conservative therapy consisting of fluids, with or without blood transfusion(s) and intensive care unit (ICU) admission for close monitoring may be sufficient [4]. If severe, then splenic artery embolization, splenic salvage, or splenectomy may be indicated when conservative management fails to achieve hemodynamic stabilization [4]. Approximately 20 to 40% of patients require surgical intervention [5]. Finally, patients should be advised to avoid high impact sports post-injury between 1 and 6 months post-rupture, depending on the degree of splenic injury [6]. Evidence for serial CT imaging to document splenic healing prior to resumption of activities is poor and is only recommended in select individuals and activities [7]. In patients post-splenectomy, there is strong evidence to encourage patients to receive pneumococcal vaccination due to reduced immunity towards encapsulated organisms [8]. Vaccination for patients undergoing nonoperative conservative management remains controversial [9].

In the following case report, we describe an atypical case of ASR secondary to an infectious etiology and the methods used to determine the cause.

## Case presentation

A previously healthy 52-year-old Caucasian woman was rushed to the emergency department after experiencing sudden acute left-sided chest pain. The pain was rated as 9 out of 10 in intensity, radiating bilaterally to her shoulders as well as in a band-like pattern across her upper abdomen and into her flanks. There was no history of preceding trauma but there was history of recent upper respiratory tract infection symptoms including fever, chills, coryza, mild pharyngitis, non-productive cough and shortness of breath 3 weeks prior, that had mostly resolved a few days before presentation.

She is employed as a communications director. She visited a local animal adoption agency 5 weeks prior to her presentation and was bitten by a stray cat. She did not receive medical attention for this bite. She has no history of immune-compromising conditions, recent travels, past travels to Africa or sick contacts. She denies any high-risk sexual or social behaviors. She does not drink alcohol but has a 30-pack year smoking history. She had no personal or family history of leukemia, lymphoma, autoimmune diseases or coagulopathies.

**Figure 1:**
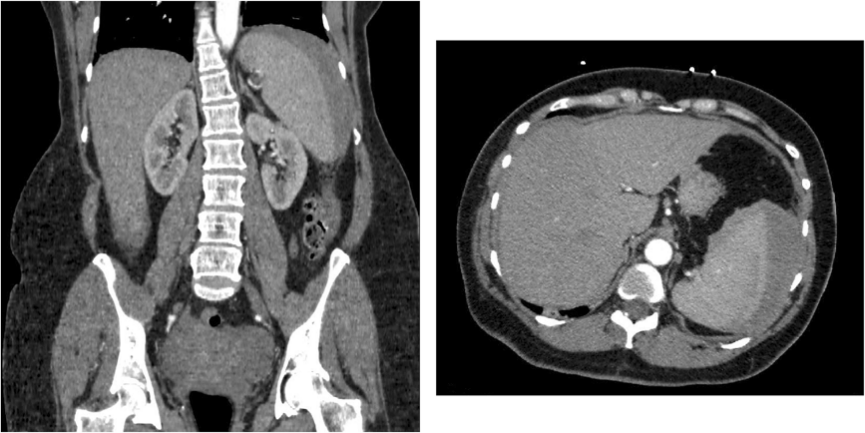
**Computed tomography images of atraumatic splenic rupture.** Representative sagittal (left) and axial (right) computed tomography images of the patient’s abdomen taken on the day she presented to the hospital. Grade 3 splenic injury (crescent-shaped subcapsular hematoma measuring up to 3cm in thickness along the lateral border of the spleen with lobulated regions of hemorrhage along the superior and medial border of the spleen and left subdiaphragmatic region, and linear densities in the spleen) and marked splenomegaly can be seen.

While in the emergency department, she was found to be alert and orientated but in significant pain. She was afebrile, heart rate of 94 beats per minute and blood pressure of 98/45mmHg. She did not have any rashes or dermatological findings. No lymphadenopathy was found but significant abdominal discomfort on light palpation and hepatosplenomegaly were noted. No rigidity or guarding or peritoneal signs were elicited. There was no rebound tenderness. CT imaging showed hepatosplenomegaly (liver span: 19.4cm; spleen length: 14.5cm) with subcapsular hematoma in the spleen and hematoma in the left subphrenic perisplenic regions, consistent with grade III splenic rupture with associated mild abdominal and pelvic ascites (Figure 1). Her liver enzymes were as follows: aspartate aminotransferase 146U/L, alkaline phosphatase 67U/L, alanine aminotransferase 166U/L and total bilirubin 6.0μmol/L. Her hemoglobin was 102g/L, white blood cells 6.8 × 10^9^ cells/L and platelets of 131 × 10^9^ cells/L. A blood smear showed no atypical lymphocytes and the differential was otherwise normal. The rest of her routine biochemical investigations were unremarkable. She received immediate hemodynamic stabilization in the ICU and was followed closely by General Surgery. During this time, she received a full infectious diseases workup. She had negative blood cultures and negative serologies for viral hepatitis, human immunodeficiency virus, cytomegalovirus, parvovirus, toxoplasmosis, and syphilis. She had negative Legionella urinary antigen. Her Monospot test was negative, but Epstein–Barr virus (EBV) serology showed EBV viral-capsid antigen (VCA) immunoglobulin (Ig) G reactivity and early antigen (EA) IgG reactivity but Epstein–Barr virus nuclear antigen (EBNA) IgG and EBV VCA IgM non-reactivity. Finally her *Bartonella* serology showed IgG reactivity (titers 1:128). Her transthoracic echocardiogram was negative for signs of bacterial endocarditis. She remained hemodynamically stable in the ICU and was discharged without need for any surgical interventions. One week after discharge, she returned to the Infectious Disease clinic with ongoing complaints of fatigue and improving abdominal pain. She was started on a 2-week course of azithromycin and rifampin and improved symptomatically (decreased pain and improved energy) and biochemically (normalization of her liver enzymes) over a 2-week period. She was seen back in follow up 3 months later. She remained well clinically with normal liver enzymes. Repeat *Bartonella* serological titers remained at 1:128 with conversion of EBNA IgG to reactive.

## Discussion

While this case concludes with the etiology of splenic rupture remaining unclear between a possible *Bartonella henselae* or an Epstein–Barr virus infection, this report has important implications for clinicians of emergency, intensive care, general surgery, hematology as well as the infectious disease medicine. As seen in Table 1, the causes of ASR are varied. A multidisciplinary approach is needed to most speedily manage and stabilize such patients. This case stands as an example of appropriate and timely management by all these departments, resulting in an excellent patient outcome requiring only conservative management.

With regards to the etiology, the patient’s symptoms and history could be explained by either *Bartonella henselae* or EBV infection. The history of a recent untreated stray cat bite, positive *Bartonella* serology and resolution of symptoms with appropriate antibiotics create a strong case for Bartonellosis, also known as cat scratch fever (CSD). However, two observations are inconsistent with this conclusion. First, in the handful of case reports noting splenic involvement in CSD, CT evidence of abscesses in the spleen or multiple scattered round hypoechoic lesions in an enlarged spleen is often, but not always, documented [10–12]. In this case, linear, not round, hypoechoic lesions were seen. The second observation that is inconsistent with a diagnosis of CSD is the relatively low anti-*Bartonella* IgG antibody titers. While the titers in this case were high enough to meet the diagnostic criteria [13], the titers persist at a lower level than expected. In the literature, typical titers are documented to be 1:1024 or greater and repeat titers 3 months later should rise [14]. However, this patient’s titers remained unchanged at 1:128. One caveat that must be kept in mind when interpreting antibody titers is that since titers are measured via serial dilutions, it is unclear where her exact antibody level is between 1:128 and 1:256. In addition, currently available *Bartonella* serological assays are poorly sensitive and there are no clear predicable kinetics of IgG or IgM titers to guide when best to retest patients for *Bartonella* serology to definitively rule in or out the infection [15]. Despite these limitations, however, given that this patient had the same titers 5 weeks post-exposure as 3 months post-exposure, this raises the possibility of a potential assay interference producing a false positive result.

A case for EBV infection is also possible given the EBV serological findings. Hepatic involvement is observed in approximately half of adults with infectious mononucleosis, resulting in up to 5-fold elevation in liver enzymes [16]. Here, liver involvement was noted clinically, radiologically and biochemically. While the patient convalesced on anti-*Bartonella* antibiotics, EBV is a self-limiting infection with a typical clinical course that resolves over 1 to 3 months. Thus, it may be possible that the improvement in clinical status had no relationship with antimicrobial therapy. A negative Monospot test is not inconsistent with an EBV infection as this test is specific, but lacks sensitivity, and has a negative likelihood ratio of 0.14 [17]. However, there are several factors in this case that argue against EBV. Infectious mononucleosis is diagnosed most commonly using the Hoagland criteria: 1) greater than 50% lymphocytes and more than 10% atypical lymphocytes; 2) clinically as a triad of fever, pharyngitis and lymphadenopathy; 3) positive serology for EBV. In this case, there were no atypical lymphocytes and her clinical presentation was not entirely consistent with the classical triad. Splenomegaly is seen in 50 to 60% of patients with infectious mononucleosis, yet splenic rupture is rare, occurring in an estimated 0.1 to 0.5% of cases and occurring almost exclusively in males [18]. Thus, this patient does not fit the profile of a high-risk patient for ASR secondary to EBV infection. Furthermore, although the patient reported two previous episodes of infectious mononucleosis in her teenage years, the EBV serological profile does not fit the categorization of either past infection or reinfection of EBV and thus is inconsistent with this history. In particular, the EBNA IgG is expected to be positive in nearly all cases of past infection or reinfection of EBV approximately 4 to 6 weeks post-initial infection, EBNA IgG titers typically rise and persist lifelong [19]. The currently used assays for EBNA are good at determining active disease as one study found an average negative likelihood ratio of 0.03 [17]. One way to address indeterminate EBV results is to perform delayed or serial serologies, which will improve accuracy. In this case, repeat EBV serologies revealed conversion of EBNA IgG from negative to positive, thus highlighting the importance of repeat testing. VCA IgG avidity testing may be helpful to distinguish primary versus past infection since there is increased binding avidity of VCA IgG during acute infection [20]. In this case, avidity testing was not conducted. Western blot and polymerase chain reaction-based analyses have been also used in the literature but currently they lack standardized operational protocols to allow for routine clinical use.

## Conclusions

This case represents a common infectious disease dilemma of appropriate clinical management in the context of discordant clinical and serological findings. Although the history of a cat bite and clinical improvement after initiation of targeted therapy against *Bartonella henselae* is consistent with the diagnosis of ASR secondary to CSD, the *Bartonella* serologies were inconclusive. The clinical presentation was atypical of EBV yet the conversion of EBNA is suggestive of the diagnosis. In such difficult cases where inconclusive results are present, the skilled clinician must always remember to prioritize treatable diseases and address the wellbeing of the patient, not the results of serologies.

Given that infections explain approximately one-quarter of ASR cases, infectious etiologies should be on the differential when a patient presents with ASR. A detailed history and physical are required to screen for infectious causes although delineating the exact infectious etiology can be challenging with the limitation of serological diagnostics.

## Methods

### EBV

A chemiluminescence immunoassay on the Abbott ARCHITECT platform was used to detect all EBV serologies. Of note, both the diffuse and restrictive forms of EA are detected via this method.

### Bartonella

IgG assay is performed via the immunofluorescence antibody assay.

## Consent

Written informed consent was obtained from the patient for the publication of this case report and accompanying images. A copy of the written consent is available for review by the Editor-in-Chief of this journal.

## Abbreviations

ASR: Atraumatic splenic rupture
CSD: Cat scratch fever
CT: Computed tomography
EA: Early antigen
EBNA: Epstein–Barr virus nuclear antigen
EBV: Epstein–Barr virus
ICU: Intensive care unit
Ig: Immunoglobulin
VCA: Viral-capsid antigen.
